# Stay@Work: Participatory Ergonomics to prevent low back and neck pain among workers: design of a randomised controlled trial to evaluate the (cost-)effectiveness

**DOI:** 10.1186/1471-2474-9-145

**Published:** 2008-10-29

**Authors:** Maurice T Driessen, Johannes R Anema, Karin I Proper, Paulien M Bongers, Allard J van der Beek

**Affiliations:** 1 Body@Work TNO VUmc, Research Center Physical Activity, Work and Health, VU University Medical Center, van der Boechorststraat 7, 1081 BT Amsterdam, The Netherlands; 2 Department of Public and Occupational Health, EMGO Institute, VU University Medical Center, The Netherlands; 3 Research Center for Insurance Medicine AMC-UWV-VUmc, The Netherlands; 4 TNO Quality of Life, The Netherlands

## Abstract

**Background:**

Low back pain (LBP) and neck pain (NP) are a major public health problem with considerable costs for individuals, companies and society. Therefore, prevention is imperative. The Stay@Work study investigates the (cost-)effectiveness of Participatory Ergonomics (PE) to prevent LBP and NP among workers.

**Methods:**

In a randomised controlled trial (RCT), a total of 5,759 workers working at 36 departments of four companies is expected to participate in the study at baseline. The departments consisting of about 150 workers are pre-stratified and randomised. The control departments receive usual practice and the intervention departments receive PE. Within each intervention department a working group is formed including eight workers, a representative of the management, and an occupational health and safety coordinator. During a one day meeting, the working group follows the steps of PE in which the most important risk factors for LBP and NP, and the most adequate ergonomic measures are identified on the basis of group consensus. The implementation of ergonomic measures at the department is performed by the working group. To improve the implementation process, so-called 'ergocoaches' are trained.

The primary outcome measure is an episode of LBP and NP. Secondary outcome measures are actual use of ergonomic measures, physical workload, psychosocial workload, intensity of pain, general health status, sick leave, and work productivity. The cost-effectiveness analysis is performed from the societal and company perspective. Outcome measures are assessed using questionnaires at baseline and after 6 and 12 months. Data on the primary outcome as well as on intensity of pain, sick leave, work productivity, and health care costs are collected every 3 months.

**Discussion:**

Prevention of LBP and NP is beneficial for workers, employers, and society. If the intervention is proven (cost-)effective, the intervention can have a major impact on LBP and NP prevention and, thereby, on work disability prevention. Results are expected in 2010.

**Trial registration:**

ISRCTN27472278

## Background

In the Netherlands the most common musculoskeletal disorders (MSD) are low back pain (LBP) and neck pain (NP) [1]. Surveys among the Dutch working population showed that the one year prevalence of LBP is 44.4% for men and 48.2% for women [2], and the prevalence of neck and shoulder pain is 28% [3]. These symptoms may lead to medical consumption [2,4], sickness absenteeism or disability claims [5-8]. In 2003, the estimated total health care costs of LBP and NP were 761 million Euros [9]. However, the annual costs of sick leave and loss of productivity due to LBP and NP are estimated to be nine times the health care cost [10]. The consequences and the costs of LBP and NP are a burden to society and companies. Therefore, prevention of these symptoms is imperative.

LBP and NP are assumed to be of multifactorial origin [11]. Several systematic reviews showed that the work-related risk factors for LBP are heavy physical workload, whole body vibration, frequent bending and twisting, and heavy (manual) lifting [12-16]. The main risk factor for NP is neck flexion [17]. High prevalence rates of LBP and NP and the presence of the risk factors in the working population indicate the need for prevention at the workplace. Workplace interventions, such as ergonomics (i.e. education on lifting techniques or postural instruction) have been frequently used. However, the evidence to recommend ergonomics for the reduction of the prevalence of LBP is not sufficient and inconsistent [18]. The evidence for preventing neck and upper extremity pain using ergonomics is also limited [19,20].

Another approach to prevent LBP and NP may be participatory ergonomics (PE). Supported by the management, PE empowers workers to design and change the worksite [21]. A recent randomised controlled trial (RCT) indicated that PE was not effective to prevent MSD among kitchen workers [22,23], whereas other studies indicated that the use of PE reduces MSD among workers [24-29]. However, most of the studies lacked a randomisation procedure, had no control group, assessed no other health outcomes (i.e. pain, quality of life, general health status, and costs), and studied homogeneous study populations only (blue or white collar) [30]. Moreover, a RCT conducted in Sherbrooke Canada, indicated that PE induced a 1.9 faster (i.e. 42 days) return to work (RTW) in patients suffering from sub acute LBP [31]. In the Netherlands, the Dutch participatory workplace intervention [32] which was derived from the Sherbrooke model [33], resulted in 30 days earlier RTW and was cost-effective when compared to usual practice [34-36].

Although PE was (cost-)effective as a RTW intervention, no RCT has been conducted to evaluate the (cost-)effectiveness of PE to prevent LBP and NP among a large and heterogeneous population of workers (blue and white collar). Therefore, the main objective of this study, called the Stay@Work study, is to evaluate the effectiveness of PE compared to usual practice (no PE) to prevent an episode of LBP and NP among workers. Secondary objectives of this study are: 1) to compare the effectiveness of PE on the secondary outcome measures (i.e. actual use of ergonomic measures, physical workload, psychosocial workload, intensity of pain, general health status, sick leave and work productivity), and 2) to evaluate the cost-effectiveness and cost-utility of PE compared to usual practice.

## Methods

### Study design

The Stay@Work study is a two-armed RCT. Workers of the departments allocated to the intervention group receive the PE programme; departments allocated to the control group receive usual practice (no PE programme). Data on all outcome measures are assessed at baseline and after 6 and 12 months. Data on the primary outcome (an episode of LBP and NP), as well as on intensity of pain, sick leave, work productivity, and health care costs are collected retrospectively every 3 months. The data collection started in November 2007.

The study protocol was approved by the Medical Ethics Committee of the VU University Medical Center. Because departments are included as a whole, the Medical Ethics Committee decided that participants did not have to sign an informed consent form.

### Study population and setting

Participants are workers, both blue and white collar workers, recruited from the departments of four large Dutch companies with at least 3,000 workers each. The companies included are a railway transportation company, an airline company, a university including its university medical hospital, and a steel company. In order to successfully accomplish a PE programme, strong management support and participation at all company levels (high, middle, low management, as well as worker level) is essential [21]. Therefore, a top-down and bottom-up strategy is applied.

Prior to the study, the company's higher management confirmed participation by signing a letter of intent and agreed that their workers at certain departments are allowed to spend working time to participate in the study. In their letter of intent, the higher management also agreed with the financial and organisational consequences of the intervention. Then, the higher management sent all managers of potential departments an information letter containing information about the study design and the intervention, and requested cooperation. The researchers informed the department managers in detail during an oral presentation, and then asked for the participation of the department. After the department manager agreed to participate, he or she informed the lower level management of the department about the study. The stakeholders involved with workers' health (i.e. human resource management, workers union, and occupational physicians) are also informed by the researchers about the study design.

Although all workers within the participating departments are invited to participate, workers have to meet the following inclusion criteria to be included in the data analyses: 1) aged between 18 years and 65 years; 2) no cumulative sick leave period longer than 4 weeks due to LBP or NP in the past 3 months before the start of the intervention; and 3) not pregnant.

### Sample size

**Figure 1:**
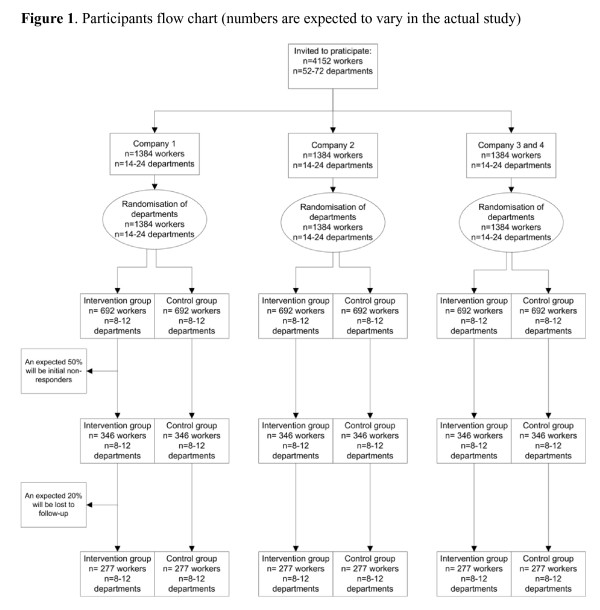
Participants flow chart

The one-year incidence of LBP and NP in a general working population are 12–14% and 6%, respectively [37,38]. However, LBP and NP are episodic in nature. Therefore, repeated outcomes assessment are performed. Based on the results of the study of IJmker et al. (2006) an intra-class correlation coefficient (ICC) of 0.73 is estimated [39]. By using the ICC, the power analysis revealed that a sample size of 1,662 workers (2 groups of 831 workers) is needed to detect a 25% decrease of an episode of LBP and NP among the intervention group compared to the control group [40]. This difference can be detected with a power of 80% and an alpha of 0.05. Expecting a dropout rate of 20% an initial study population of 2,076 workers is needed (see figure 1).

### Randomisation

Each department consists of approximately 150 workers. If necessary, to obtain a 'department' size of approximately 150 workers, departments are clustered to one department using the revised version of the Dutch Classification of Occupations 1984 (e.g. mentally demanding work, mixed mentally or physically demanding work, light physically demanding work, and heavy physically demanding work) [41]. All departments are pre-stratified using this classification. Randomisation is performed at the level of department, in order to avoid contamination from workers allocated in the intervention to those in the control group. Using a computer-generated randomisation (Random Allocation Software, version 1.0, May 2004, Isfahan University of Medical Sciences, Iran), the randomisation is performed by an independent researcher (e.g. research assistant), who has no prior information about the departments. For practical reasons, the randomisation is performed before baseline measurements.

### Blinding

Obviously, as a result of the PE intervention it is impossible to blind researchers, ergonomists, and department managers. However, workers of the departments randomised to the intervention or the control group are not aware of the study design. Only the department managers are informed about the study design and the randomisation outcome and are asked not to communicate to workers about the study design. Moreover, to further blind the workers for the study design, both groups watch a movie with ergonomic instructions which is used as a sham intervention.

### Study groups

#### Control group

To the workers allocated to the control departments are asked to watch three short (45 seconds) web-based educative movies about the prevention of LBP and NP at the campaign website of 'Lighten the load, a European Campaign on Musculoskeletal Disorders' developed by the European Agency for Safety and Health at Work. The movies show certain risk factors at work (i.e. lifting too heavy loads, frequent twisting of the lower back, holding the neck in a fore ward bent position for a prolonged time) for LBP and NP as well as (ergonomic) strategies to avoid these risk factors and, thereby, prevent LBP and NP. The movie is used as a sham intervention and is considered as a educative strategy, which showed to be ineffective to prevent LBP [42].

#### Intervention group

Workers allocated to the intervention departments watch the same movies about the prevention of LBP and NP as the control group. In addition, they receive the Stay@Work PE programme (see below).

### Intervention

One of the main characteristics of PE is the formation of a 'working group' in which both workers and management participate as members [21,43]. The six steps of the Stay@Work PE programme are followed during two meetings with the working group. The first working group meeting is obligatory, and the second meeting is optional. The first meeting is guided by an ergonomist. During a six hour training session, which was held one month before the start of the intervention and consisted of a theoretical and a practical part, the participating ergonomists are trained in the protocol.

Each working group is formed by the department manager of each intervention department and consists of a maximum of 10 members; each member has his or her own role during the working group meetings. The working group includes:

- Eight workers who are representatives for the main job tasks performed at the department, who have worked for at least 2 years in the current job, and who work more than 20 hours per week at the department. Workers have to identify risk factors for LBP and NP and have to define adequate ergonomic measures for these risk factors.

- One department manager (or a representative) having decision authority and who know whether the ergonomic measures suggested are feasible on organisational and financial criteria.

- One occupational health and safety (OHS) coordinator who judged to what extent the ergonomic measures fit in the health and safety policies measures.

After forming the working group, the researchers plan a date for the first and second working group meeting and instruct the working group in the six steps of PE and their specific roles during the meeting. In case a member of the working group is unable to attend the working group meeting him or herself, the department manager selects and asks a substitute. If the department manager or the OHS coordinator is not able to attend the working group meeting, a representative is asked to take their place.

The Stay@Work PE programme consists of the following six steps:

#### Step 1 The inventory of the workplace

As part of the preparation of the first working group meeting, an inventory of the workplace is conducted one month prior to the meeting consisting of the following sub steps:

1. Pictures of risk factors for LBP and NP are made: each worker of the working group is equipped with a photo camera and is instructed to take at least 10 pictures of risk factors for LBP and NP at the worksite.

2. Data of all workers of the department are obtained from the baseline questionnaire, and is used to obtain information on psychosocial risk factors for LBP and NP present at the department

3. The ergonomist conducts a worksite observation at the department by using a checklist. The ergonomist observes activities relevant for LBP and NP at work (e.g. type of work performed, lifting heavy loads (> 20 kilograms), frequent bending and rotating the lower back or neck). Furthermore, the ergonomist collects information about co-worker support, job organisation, job planning, instructions, skills, management styles, materials, and equipment.

According to a fixed format, all information is summarised in a document by the research assistant for each department, and serves as a starting point for the first working group meeting. One week before the first working group meeting, the document is sent to the ergonomist and all members of the working group.

In the first meeting lasting six hours, the working group follows steps 2–4 of the Stay@Work PE programme. The meeting is guided by the ergonomist and takes place in one of the regular conference rooms of the department.

#### Step 2 Analysis of risk factors

All members of the working group discuss and if necessary adjust risk factors for LBP and NP summarised in the document, and a brainstorm session is performed to add possible other risk factors (individual, physical, mental, and organisational). Then, the frequency and the severity of the risk factors is evaluated by rating them according to a criteria list. The most frequent and severe risk factors are written down on a flap-over and are prioritised by all members of the working group. Subsequently, each member of the working group is asked to award his or her three most important risk factors by adding a sticker. On the basis of consensus, the three risk factors with the highest number of stickers are considered as the three most important risk factors.

#### Step 3 Finding of ergonomic measures

According to the nominal group technique [32] the working group performs a brainstorm session about different types of ergonomic measures (individual, physical, mental, and organisational) to reduce the prioritised risk factor. The ergonomic measures are evaluated using a criteria list, considering the problem solving capability, costs, compatibility, complexity, and feasibility of the ergonomic measures [44]. The manager decides whether the costs for the ergonomic measures are feasible. Furthermore, the ergonomic measures are judged whether they can be implemented within three months. Prioritisation of the ergonomic measures is performed similarly to step 2, resulting in the three most adequate ergonomic measures on the basis of consensus.

#### Step 4 Preparation of an implementation plan

The working group writes down the prioritised three most adequate ergonomic measures for the three most important risk factors for LBP and NP in an implementation plan. The plan describes who is responsible for the implementation of the ergonomic measures; what type of activities need to be performed by who, how, and when a test phase is needed; and whether an appointment for a second meeting to evaluate the implementation plan is required (see step 6). After finishing the first meeting, all members of the working group receive a copy of the implementation plan.

#### Step 5 Implementation of ergonomic measures

In the weeks following the first meeting, the working group informs the co-workers about the ergonomic measures, motivates and instructs them on how to use the ergonomic measures. The OHS coordinator or the department manager is the central person for coordinating and facilitating the implementation process. Studies on PE report difficulties towards the implementation of ergonomic measures [25] and the actual use of ergonomic measures [45]. Therefore, to further improve the implementation process and the actual use of the ergonomic measures, two or three workers are trained to be a 'Stay@Work ergocoach'. During a four hour training session, they are instructed about implementation strategies that can be used to inform, motivate, and instruct the co-workers about the selected ergonomic measures, and to learn how to deal with co-workers' resistances against the ergonomic measures. At the end of the training session they receive the 'Stay@Work ergocoach toolkit', which includes formats of e-mails, posters, flyers, and digital presentations. The toolkit is used as an instrument to inform the co-workers at the department about the prioritised ergonomic measures.

#### Step 6 Evaluation and control of the ergonomic measures

In step 4, the working group decides whether the second meeting (one hour) is needed to evaluate the status of the implementation plan or the test phase. The ergonomist does not attend the second meeting, unless he or she is asked by the working group. The rationale is that the implementation should be the responsibility of the department and the working group.

### Use of co-interventions

In both the intervention group and the control group, the use of co-interventions are registered. Using a questionnaire, the department managers are asked about all other ongoing studies, planned reorganisations and other innovations or company health interventions (i.e. fitness programmes, back schools, chair massages, and lifestyle programmes).

### Data collection procedure

Depending on the availability of an e-mail account supported by the company, outcome measures are collected either by online questionnaire or by hard copy questionnaires. If companies prefer online questionnaires, an e-mail is sent to the workers containing a link to the online questionnaire. If companies prefer hard copy questionnaires, the questionnaires are sent to the department managers, who hand out the questionnaires to the workers. The completed questionnaires are collected by the researchers. Approximately, one month before the first working group meeting, all workers of the intervention departments of concern and those of the matched control departments, receive the baseline questionnaire. To reduce loss to follow-up, a maximum of three reminders are sent and each department manager is asked to encourage all workers to complete the questionnaires. Subsequently, at each measurement, the researchers visit the participating departments before, during baseline and during follow-up measurements to encourage workers to fill out their questionnaires. Additionally, incentives (e.g. gift vouchers and pie) are used.

### Primary outcome measure

#### An episode of LBP and NP

Every 3 months, the primary outcome measures, an episode of LBP and NP, are assessed using a modified version [46] of the Nordic Questionnaire [47]. LBP and NP are episodic and recurrent. This implies that one may have more than one episode of LBP and NP during follow-up. An episode of LBP and NP is defined by the presence of LBP and NP during a recall period of 3 months followed and preceded by a recall period of 3 months without LBP and NP. The transition from a symptom free period to a new episode of LBP and NP is modelled as the outcome variable.

### Secondary outcome measures

#### Actual use of ergonomic measures

After 6 and 12 months, the researchers monitor whether the ergonomic measures are implemented or not, and classify the ergonomic measures according to the Stapleton classification scheme for ergonomic measures [48]. It is known that the actual use of ergonomic measures is positively and significantly associated with behavioural change phases [49]. Therefore, the behavioural determinants Attitude-Social influence- self-Efficacy (ASE) [50] needed to measure determinants for (the intention to perform) the desired behaviour (actual use of ergonomic measures) are asked using five questions at baseline, after 6 months and 12 months.

#### Physical workload

Data concerning the physical workload is obtained from the Dutch Musculoskeletal Questionnaire (DMQ) [46]. Proven physical risk factors are assessed: heavy physical workload, whole body vibration, frequent bending and twisting, and heavy (manual) lifting [12-16] for LBP, and neck flexion [17] for NP.

#### Psychosocial workload

Data on psychosocial workload are assessed by means of a Dutch version of the Job Content Questionnaire [51] using the following indices: skill discretion, decision authority, psychosocial job demands, supervisor support, and co-worker support. These indices have shown moderate to good reliability (0.65–0.81) [52]. The psychosocial stressors and perceived stress are assessed using the 11-item 'need for recovery scale' from the Dutch version of the Questionnaire on the Experience and Evaluation of Work (Dutch abbreviation VBBA), which has shown to be valid and reliable (0.86) [53,54].

#### Intensity of pain

The intensity of pain (i.e. pain at the moment of filling out the questionnaire, average pain and most severe pain experienced in the past 3 months), and the pain duration (total days of pain experienced in the past 3 months) due to LBP and NP is measured using von Korff scales, which have shown acceptable to good test-retest reliability [55,56].

#### General health status

The Dutch version of the EuroQol is used to assess the patient's general health status. The questionnaire describes the general health status in five dimensions: mobility, self-care, usual activities, pain/discomfort and anxiety/depression [57]. Furthermore, one question is adopted from the Dutch ShortForm-36 questionnaire, which has shown satisfactory validity and reproducibility [58].

#### Sick leave and work productivity

Self-reported all cause sick leave is measured using a single item question asking the workers about their full days of absence from work due to sick leave in the past 3 months. The same question is used to assess sick leave due to LPB or NP in the past 3 months. These questions have shown acceptable specificity and sensitivity levels [59]. Additional data on days of sick leave and diagnoses are collected from the records of the Occupational Health Service and Human Resource department of the participating companies. Work productivity is measured using a single item question from the WHO Health Productivity Questionnaire [60,61] asking participants to report their overall work productivity on a 10-point scale in the past three months.

### Other variables

#### Sociodemographic

At baseline, sociodemographic data, (i.e. age, gender, level of education, working days per week, working hours per week, nationality, body height, and body weight) are assessed using the DMQ [46].

#### Physical Activity

Lack of physical activity might be a risk factor for LBP and NP [62,63]. Therefore, physical activity (during work, sports, during other leisure-time pursuit, and in total) is assessed using the Baecke questionnaire [64,65], which has shown acceptable reliability and validity [66].

### Cost data

A cost-effectiveness analysis (CEA) and a cost-utility analysis (CUA) of the Stay@Work PE programme is performed. The CEA is performed using both a company and a societal perspective. The company perspective compares the intervention costs paid by the company with 1) the effect on the prevalence of LBP and NP; 2) the effect on sick leave (in days) [67]; and 3) work productivity. Intervention costs include costs for the development of the intervention, the implementation of the intervention (i.e. materials needed for the working group meetings, the Stay@Work ergocoach training, and costs of the ergonomists).

Next to the costs relevant for the employer, the societal perspective takes into account all costs (i.e. direct and indirect costs, and costs within and outside the health care). Direct health care costs include costs of the visits to health care providers, diagnostic examinations, and prescribed medication due to LBP and NP. Direct non-health care costs are costs outside the formal health care system due to LBP and NP and include costs of the ergonomist, time loss of workers in the working group, and over-the-counter medication. Both direct health care cost and direct non-health care cost are measured every three months by using retrospective cost questionnaires [68,69]. The indirect non-health care costs are the costs of production losses due to sick leave, reduced productivity while at work, and work disability of the worker. The CUA estimates the incremental costs per Quality Adjusted Life Year. Utilities are measured by the EuroQol.

### Process evaluation

The process of the intervention is evaluated in four ways:

First, the working group is asked for their opinions on: 1) the content and process of the working group meeting as a whole; 2) the ergonomist's competences; and 3) their expectations towards the implementation and the effectiveness of the ergonomic measures on the prevention of LBP and NP.

Second, working group members who followed the Stay@Work ergocoach training are asked their opinions about: 1) the training as a whole; and 2) the added value of the training to improve the implementation process and to improve the actual use of ergonomic measures.

Third, all workers of the intervention and control departments are asked: 1) if they are aware of prioritised ergonomic measures and whether the ergonomic measures are implemented at the department; 2) if they actually use the ergonomic measures; and 3) about the perceived effectiveness of the implemented ergonomic measures on LBP and NP prevention.

Fourth, all members of the working group are sent a questionnaire and are asked: 1) whether he or she implemented the ergonomic measure(s) for which he or she is responsible; and 2) to identify and describe possible barriers or facilitators during the implementation of the ergonomic measure(s). One worker of the working group is invited for a semi-structured interview in which the implementation process is discussed. The content and structure of the interview is based on the answers given in the questionnaires of all working group members. Furthermore, the manager is sent a questionnaire and is also invited for a semi-structured interview.

### Statistical analyses

All analyses are performed according to the intention to treat principle. The most important analyses are performed at worker level. Two analyses are performed: (1) a crude analysis with the outcome variable measured at follow-up as the dependent variable adjusted for the outcome, measured at baseline, and (2) an analysis as above but adjusted for potential covariates (e.g. gender, age, type of work, history of LBP and NP, and physical and psychosocial workload). Effects of the intervention will be checked for effect modification (gender, type of work, number of ergonomic measures implemented). For the purpose of primary prevention a subgroup analysis is performed among workers without LBP and NP in the month prior to the start of the intervention [70]. Generalised estimation equations (GEE) are used to analyse long-term results (i.e. 12 months after baseline) and to investigate the transition of no episode to an episode of LBP and NP during a 3-month period. Furthermore, analyses at department level are performed by the use of multilevel analysis. For all analyses a two-tailed significance level of <0.05 is considered statistically significant. The multilevel statistical analyses are performed with MlwiN 2.0; linear and logistic regression analyses is performed with SPSS 14.0 (SPSS Inc. Chicago, Illinois, USA), and GEE analyses is performed with STATA version 7.0, College Station, TX).

### Cost-effectiveness analysis

The indirect costs for production losses due to sick leave are calculated by using the Friction costs method [71]. For this method, the Dutch guidelines for economic evaluation is used [72]. The direct health care costs are calculated by using tariffs for the costs of health care professionals and market prices for the value of medication. Costs for the ergonomists are calculated by using the hourly wages. The direct non-health care costs, are calculated by using the information obtained from the cost questionnaires and shadow prices. Bootstrapping is used for comparison of mean direct, indirect and total costs between the two groups. Confidence intervals are obtained by bias corrected and accelerated bootstrapping. Cost-effectiveness ratios are calculated by dividing the difference between the mean costs of the interventions by the difference between the mean effects of the interventions. The bootstrapped costs effects pairs are graphically presented on a cost-effectiveness plane. Acceptability curves are calculated in order to show that the probability of the intervention is cost-effective at a specific ceiling ratio. Furthermore, sensitivity analyses are performed.

## Discussion

Prevalence of LBP and NP among Dutch workers is high and the financial consequences are a considerable burden to companies and society [2,3,9]. In previous studies PE has been applied to prevent MSD; however, most studies lacked a randomisation procedure or a control group. One of the main strengths of Stay@Work is that this study is one of the few RCT's that evaluates PE aimed at the prevention of an episode of LBP and NP. Moreover, this study evaluates the (cost-)effectiveness of PE, and investigates other important health outcomes among a large heterogeneous population of workers. To date, research populations are consisting of construction workers, cleaners, glaziers, and manufacturing workers. In this study also health care workers, industrial and white collar workers are studied. A second strength is that the participants are blinded to the study design and the randomisation outcome, which minimises the chance that they undertake actions that may interfere with the experimental study design. A third strength is the use of an appropriate implementation strategy. Van der Molen et al. (2005) reported that the use of facilitation and educational strategies in the implementation of ergonomic measures lead to higher completed behavioural change phases and increased use of ergonomic measures [73]. This is confirmed by Jensen and Friche (2008), who used an implementation strategy that increased the use of ergonomic measures and successfully reduced severe knee problems among floor layers [26]. To our knowledge, this is the first study that trained ergocoaches to improve the implementation of the ergonomic measures and stimulate the co-workers to use the ergonomic measures. A fourth strength is that Stay@Work evaluates the effectiveness of PE under routine department circumstances and does not optimise the study conditions (i.e. stopping with co-interventions). In other words, it is an effectiveness study and not an efficacy study.

There are also some limitations. First, selection bias due to a selective response may occur. Workers with LBP and NP could be more likely to fill out the questionnaires compared to workers without complaints. Second, due to the maximum size of the working group (10 persons), the department manager selects representatives of the largest and most important task groups to participate in the working groups. Therefore, very small task groups may not be represented in the working group. The ergonomic measures are developed for the department as a whole, consequently, the non-representation of the smallest task groups might lead to a lower actual use of the ergonomic measures among workers from these groups. Third, although the randomisation and the deliverance of the intervention are carried out at the level of the department, the main statistical analyses are performed at worker level. However, based on the example described in the book, we expect that by using multilevel analysis the differences and equalities between the analyses performed at department level and analyses performed at worker level are comparable to the differences of studies in which the randomisation was carried out at worker level [74].

Studying the effects of this intervention is important, as it aims to prevent a major occupational health problem. If proven (cost-)effective, the companies will benefit from a bottom-up method to prevent LBP and NP among their workers. Occupational Health Services or managers may incorporate this method in their usual prevention management.

## Competing interests

The authors declare that they have no competing interests.

## Authors' contributions

All authors contributed to the design of the study. MTD is the principle researcher and is responsible for the data collection. JRA contributed to the conception and the design of the study and coordinates the study. JRA, KIP, PMB, and AJvdB supervise the study. JRA and AJvdB act as the guarantors of the study. All authors contributed to writing up of this paper and approved the final manuscript.

## Pre-publication history

The pre-publication history for this paper can be accessed here:


